# Changes in the Quality of High-Oleic Sunflower Oil during the Frying of Shrimp (*Litopenaeus vannamei*)

**DOI:** 10.3390/foods12061332

**Published:** 2023-03-21

**Authors:** Jiechang Chen, Yuanyuan Zhao, Runlin Wu, Tao Yin, Juan You, Benlun Hu, Caihua Jia, Jianhua Rong, Ru Liu, Binjia Zhang, Siming Zhao

**Affiliations:** 1 Key Laboratory of Environment Correlative Dietology (Ministry of Education), College of Food Science and Technology, Huazhong Agricultural University, Wuhan 430070, China; 2 Chongqing Key Laboratory of Speciality Food Co-Built by Sichuan and Chongqing, College of Food Science, Southwest University, Chongqing 400715, China

**Keywords:** high-oleic sunflower oil, frying, *Litopenaeus vannamei*, ^1^H NMR, oxidation products

## Abstract

Shrimp fried in vegetable oil is a very popular food, so it is important to study the changes in the quality of the oil during frying. In order to more precisely study the nature of frying oil during the cooking process, this study investigated the quality changes of high-oleic sunflower oil during the frying of South American white shrimp (*Litopenaeus vannamei*). The oxidation and hydrolysis products of frying oils were investigated by integrating the proton nuclear magnetic resonance technique with traditional oil evaluation indexes in an integrated manner. The results showed that the color difference as measured using the ΔE* value increased gradually during the process. Moreover, the acid value, carbonyl value, and total oxidation significantly increased with prolonged frying time. The major oxidation products formed during frying were (*E,E*)-2-alkenals, (*E,E*)-2,4-alkadienals, and *E,E*-conjugated hydroperoxides. This indicated that longer treatment times corresponded with an increased accumulation of aldehydes and ketones, and an increased degree of oxidative deterioration of the oil. However, the proportion of oleic acid in the frying oil increased with the frying of shrimp, reaching 80.05% after 24 h. These results contribute to our understanding of the oxidative deterioration of high-oleic oils during frying, and provide an important reference for the application properties of high-oleic oils.

## 1. Introduction

Frying is a traditional food processing method that can improve the color and taste of food, give it unique flavors [1], and preserve it for a longer time [2]. However, during the frying process, the oil undergoes a series of chemical reactions, such as oxidation, hydrolysis, isomerization, and polymerization, under the influence of factors such as high temperature, oxygen, and moisture [3]. This leads to a change in color, increase in viscosity, and reduction in quality, and can even produce substances harmful to humans, including aldehydes, acids, ketones, alcohols, and others such as *trans* fatty acids, triglyceride polymers, acrylamide, and so on [4]. In addition, most commercially produced fried foods are fried in batches in the same oil without changing the oil frequently. Fried food batch production is more likely to deteriorate oil into harmful substances [5]. The breakdown products created during prolonged, high-temperature deep-frying include free fatty acids, glycerol esters, oxidized glycerol esters, polymerized glycerol esters, and other polar components [6]. Fried meals absorb the majority of these dangerous compounds, and many aldehydes, such as secondary oxidation products, are cytotoxic and neurotoxic and can harm the human body. Alkenes with 4-hydroxy-(*E*)-2-alkenals and 4-hydroperoxy-(*E*)-2-alkenals groups generated via oil oxidation may lead to disorders such as autoimmune diseases, Alzheimer’s disease, cancer, and cardiovascular disease in humans [7,8].

High-oleic sunflower oil has good oxidative stability, high-quality fatty acid composition [9], and is especially rich in oleic acid, which is an unsaturated fatty acid that has higher oxidative stability than other unsaturated fatty acids [10]. Oils rich in oleic acid are more resistant to degradation and produce smaller amounts of toxic aldehydes than those rich in linoleic and linolenic acids [11]. Furthermore, oils rich in oleic acid induce oxidative rancidity over their shelf life or decompose slowly during frying [12]. Furthermore, consuming oleic-acid-rich oils has several health benefits, such as decreased cholesterol level in the blood and reduced cholesterol deposition in blood vessels, and enhanced high-density lipoprotein level, which can improve cardiovascular health [13]. However, Sayyad et al. [14] discovered that the amount of linoleic acid in high-oleic sunflower oil has a significant impact on its frying effectiveness. Therefore, further research is required to evaluate the frying performance of high-oleic sunflower oil. In this study, the changes in high-oleic sunflower oil during the frying of South American white shrimp (*Litopenaeus vannamei*) were monitored. The oxidation and hydrolysis products of high-oleic sunflower oil were analyzed using proton nuclear magnetic resonance (^1^H NMR). Several conventional physicochemical indicators, such as acid value (AV), carbonyl value (CV), and total oxidation (TOTOX), were also analyzed. The aim of this study was to investigate the oxidation process of high-oleic sunflower oil while frying *L. vannamei* shrimp, which will contribute to a more comprehensive understanding of the application properties of high-oleic oil and the effect of shrimp on the quality of frying oil.

## 2. Materials and Methods

### 2.1. Materials and Chemicals

*L. vannamei* shrimp (Huazhong Agricultural University Agricultural Products Market, Wuhan, China), and high-oleic sunflower oil (Zhongliang Co., Ltd., Beijing, China).

Anhydrous ethyl ether, isopropyl alcohol, potassium hydroxide, chloroform, methanol, potassium thiocyanate, ferrous chloride, *p*-anisidine, hydrochloric acid, and glacial acetic acid were purchased from Sinopharm Chemical Reagent Co., Ltd. (Shanghai, China). Isooctane, 2,4-dinitrophenylhydrazine, and phenolphthalein were from Shanghai Macklin Biochemical Co., Ltd. (Shanghai, China). Deuterated chloroform (CDCl_3_) was acquired from Cambridge Isotope Laboratories Inc. (Andover, MA, USA).

### 2.2. Sample Preparation

*L. vannamei* shrimp were thawed overnight at 4 °C, washed, deshelled, and deveined. Tails were left and 90% of the back was cut after grading. They were rinsed at 10 °C, and the amount of water used was controlled.

**Figure 1:**
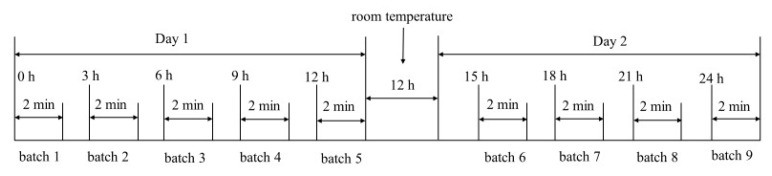
Sampling method used for oil samples in frying experiment.

Frying experiments were performed in a DF-6L deep-fryer (Jieguan Co., Ltd., Dongguan, China). For the frying trials, two frying pots were used, each with 6 L of high-oleic sunflower oil. One pot was used for the fried shrimp group, while the other was used for the control (no shrimp were added and the oil was heated at a constant temperature). The temperature was controlled using an infrared temperature device (Xima Co., Ltd., Zhaoqing, China) and heated to 180 ± 5 °C within 10 min. In order to maintain stable quality and heat transfer efficiency, the solid-to-liquid ratio was adjusted to 1:15 (kg:L); the details are shown in Table 1. A batch of pretreated shrimp was fried for 2 min every 3 h, 150 mL oil was sampled from both pots before and after each batch, and no fresh oil was added to the fryer. We used one frying pot to fry three times. The samples were stored at −80 °C for further analysis. The specific frying process is shown in Figure 1.

### 2.3. Determination of Moisture and Fat Content

Moisture and fat content were determined according to AOAC official methods 950.46 and 960.39 [15], respectively.

### 2.4. Determination of Oil Color

The oil color was determined according to the method described by Bansal et al. [16], with minor modifications. The colorimetric chromaticity of the samples was determined quantitatively using an automatic colorimeter (TA Instruments Corp, Newcastle, DE, USA). The colorimetric spot diameter was 10 mm and a white plate was used as the standard sample. The measured L*, a*, and b* values indicate the chromaticity of the fried oil samples, and L_0_*, a_0_*, and b_0_* represent the chromaticity of fresh oil. The ΔE* values were calculated using the following equation:

(1) $$\Delta E^{*}=\sqrt{{\left(L_{0}^{*}-L^{*}\right)}^{2}+{\left(a_{0}^{*}-a^{*}\right)}^{2}-{\left(b_{0}^{*}-b^{*}\right)}^{2}}$$

where L* is lightness, a* is redness/greenness, b* is yellowness/blueness, and ΔE* is color difference.

### 2.5. Determination of Acid Value (AV)

The AV of the oil was determined according to AOCS Official Methods Cd 3d-63 [17], and expressed as mg KOH g^−1^.

### 2.6. Determination of Carbonyl Value (CV)

The CV of the frying oil was determined according to the method described by Farhoosh et al. [18], with minor modifications. Frying oil (0.05–0.5 g) was dissolved in 10 mL of chromatographically pure isopropanol and 1 mL was taken out and placed in a 15 mL centrifuge tube to which 1 mL of 2,4-dinitrophenylhydrazine reaction solution was added. The tube was covered and shaken well, and heated in a water bath at 40 °C for 30 min, along with a blank test. After the reaction, the mixture was removed and cooled down to room temperature (25 °C) with running water, 8 mL of 2.5% KOH–isopropanol solution was added, the mixture was centrifuged at 3000 rpm for 5 min, and the absorbance of the supernatant was measured at 420 nm.

(2) $$CV=\frac{0.8291\times (A-A_{0})\times 1000}{854\times \frac{V_{2}}{V_{1}}\times m}$$

where *A* is the absorbance of the sample, *A*_0_ is the absorbance of the blank, V_1_ is the total volume after sample dilution, V_2_ is the volume of the sample dilution used for the determination, and m is the sample weight.

### 2.7. Determination of Total Oxidation (TOTOX) Value

The peroxide value (PV) of the oil was determined using the method described by Shantha et al. [19]. The oil sample (0.1–0.3 g) was dissolved in 10 mL of chloroform–methanol (7:3) mixture, 50 μL of potassium thiocyanate solution was added and vortexed for 2–4 s, 50 μL of FeCl_2_ solution was added and vortexed for 2–4 s, and then the sample left to react for 5 min at room temperature. The absorbance of the solution was measured at 500 nm (the measurement operation was completed within 10 min).

The PV was calculated using the following equation:

(3) $$PV=\frac{(A_{s}-A_{b})\times 41.25}{55.84\times m_{0}\times 2}$$

where *A_s_* is the absorbance of the sample, *A_b_* is the absorbance of the blank, and m_0_ is the weight of the sample.

The *p*-anisidine values (*p*-AV) of oils and fats are determined with reference to the AOCS Official Method Cd 18–90 [20]. The oil sample (0.5–4.0 g) was added to a 25 mL volumetric flask, dissolved with isooctane, and diluted to scale as an oil solution. The absorbance of the solution was measured at 350 nm, and isooctane was used as a blank control and recorded as *A_b_*. The oil solution (5 mL) and *p*-anisidine reagent (1 mL) were mixed well to create the test solution. After 10 min, absorbance was measured at 350 nm and recorded as *A_s_*.

The *p*-AV was calculated using the following equation:

(4) $$p\text{-}AV=\frac{25\times (1.2A_{s}-A_{b})}{m}$$

where *A_s_* is the absorbance of the test solution that reacts with the *p*-anisidine reagent, *A_b_* is the absorbance of the blank control, and m is the weight of the oil sample.

After measuring the PVs and *p*-AVs, the TOTOX values of the oil samples were calculated using the following equation [21]:

(5) TOTOX=2PV+p-AV

### 2.8. Proton Nuclear Magnetic Resonance (^1^H NMR) Spectroscopy

Oil samples from the control and deep-fried shrimp groups were taken for ^1^H NMR spectrometry analysis every 6 h during the frying process. The ^1^H NMR spectra of the frying oil were recorded according to the method described by Jia [22]. Briefly, 50–100 mg of the oil sample was dissolved in 0.6–0.7 mL CDCl_3_ and vortexed for 30 s. The mixture was then transferred to an NMR tube for further analysis. The measurement conditions were as follows: 12,335.5 Hz spectral width, 16 scans, 2.656 s acquisition time, and 25 °C.

Changes in oleic, linoleic, linolenic, and saturated fatty acids in high-oleic sunflower oil during the frying of South American white shrimp were determined using ^1^H NMR. These were calculated according to the equations described by Guillén et al. [23].

### 2.9. Statistical Analysis

All experiments were performed in triplicate, and the data were expressed as mean ± standard deviation using Origin 9.0 software (OriginLab Corp, Northampton, MA, USA). ^1^H NMR data were analyzed using MestReNova software. SPSS 23 software (IBM Corp, Armonk, NY, USA) was used to perform analysis of variance (ANOVA) and Duncan’s test (*p* < 0.05).

## 3. Results and Discussion

### 3.1. Components of Litopenaeus vannamei

The fat content of the *L. vannamei* shrimp was 2.80% and the moisture content was 72.88%. The presence of moisture accelerates the oxidation of oil [24]. Additionally, *L. vannamei* shrimp contain about 10–40 mg/kg of astaxanthin [25]. Astaxanthin is a substance with strong antioxidant capacity, which is of great application value in production. It may migrate into the oil during the frying process, thus retarding the oxidation of the oil [26].

### 3.2. Effect of Frying Time on the Color of Frying Oil

**Figure 2:**
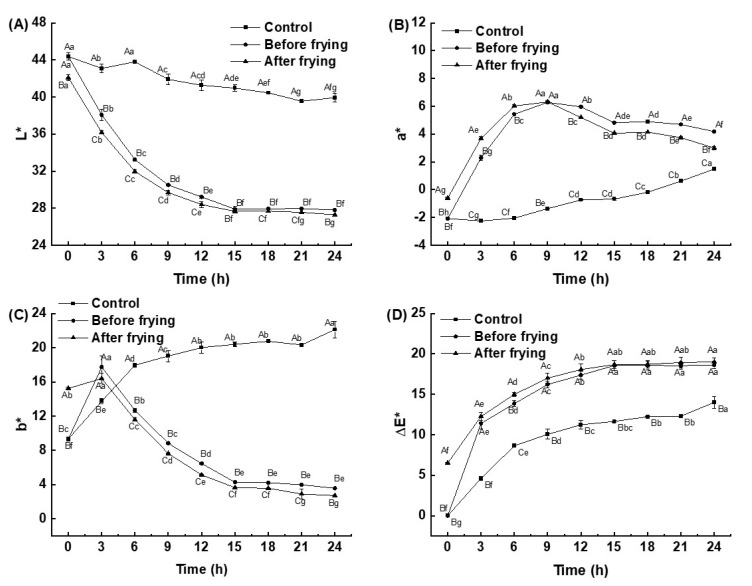
Changes in L* values (**A**), a* values (**B**), b* values (**C**), and ΔE* values (**D**) of high-oleic sunflower oil after different frying times. Note: Different lowercase letters indicated that there were significant differences among the samples prepared with the same deep-frying treatment and different frying times (*p* < 0.05), and different capital letters indicated that there were significant differences among the samples prepared with the same deep-frying time and different deep-frying treatments (*p* < 0.05).

Chroma is an important physical index of frying oil which can reflect the changes in oil quality more intuitively [27]. Figure 2 shows the chroma change of high-oleic sunflower oils with different heating times. With the prolongation of heating time, the L* value of the frying oil first significantly reduced and then stabilized; this may have been because the oil gradually deteriorated, transparency decreased, and color gradually changed from light yellow to dark brown. The L* values of the deep-fried shrimp group were much lower than those of the control because some fat-soluble substances in the *L. vannamei* shrimp migrated into the oil during the frying process, which increased the turbidity and viscosity. The a* value of the control increased with the extension of heating time, while the a* values of the deep-fried shrimp group first increased and then decreased. The increase was due to the Maillard reaction that occurred during the frying process [28], which also led to the reddening of the oil. The decrease of the a* values in the late stages of frying may have been because some substances in the shrimp migrated and reacted with the oil. The b* values of the deep-fried shrimp group first increased and then decreased to stabilization, and were lower than that of the unheated fresh oil. This indicates that the high-oleic sunflower oil gradually turned greenish during the frying process. The ΔE* values of the frying oil showed a trend of increasing and then stabilizing with increasing heating time, and the rate of increase for the deep-fried shrimp groups was much higher than that of the control, which may have been due to the decomposition of oil during the high-temperature frying process to produce carbon-based compounds. The increase in carbon-based compounds led to a darker color and increased ΔE* values [29].

### 3.3. Effect of Frying Time on the AV and CV of Oil

**Figure 3:**
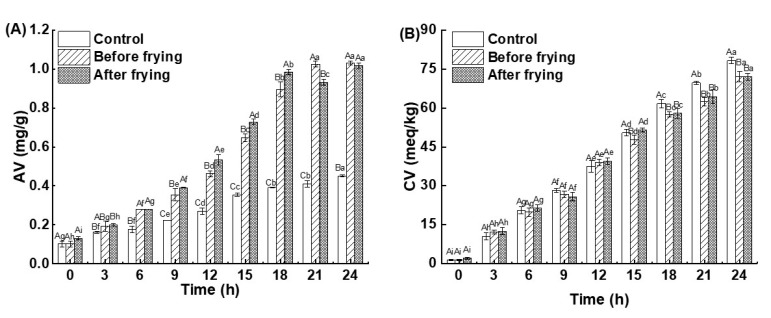
Changes in acid values (AVs) (**A**) and carbonyl values (CVs) (**B**) of high-oleic sunflower oil after different frying times. Note: Different lowercase letters indicated that there were significant differences among the samples prepared with the same deep-frying treatment and different frying times (*p* < 0.05), and different capital letters indicated that there were significant differences among the samples prepared with the same deep-frying time and different deep-frying treatments (*p* < 0.05).

As a measure of the degree of hydrolysis and rancidity of oil, AV can represent the amount of free fatty acids present in an oil [29]; generally, the smaller the AV, the better the quality of the oil. Figure 3A shows the changes in the AVs of the high-oleic sunflower oil at different frying times. It can be seen that the AVs of the control were increased significantly in both before and after frying groups. This may be related to the generation of free fatty acids through the thermal oxidation and hydrolysis of the frying oils. The longer the frying time, the higher the degree of oil oxidation and hydrolysis, and the greater the AV [14]. The AVs of the deep-fried shrimp group were much higher than those of the control because the water and fat from the *L. vannamei* shrimp partially migrated into the frying oil, accelerating hydrolysis and changing the fatty acid composition and saturation of the oil, resulting in a relative increase in the free fatty acid content, which subsequently led to an increase in the AV [30,31]. Furthermore, the sampling method of keeping a constant solid-to-liquid ratio resulted in a significant shift in the ratio of surface area to volume of oil, which could have been a factor in the increase in the AV of the frying oil.

The CV refers to the total quantity of fatty acids, glycerides, and the polymers made up of aldehyde and ketone groups that are produced as a consequence of the decomposition of peroxides, which give fried foods an unpleasant taste and lower their nutritional value [6]. Figure 3B displays the change in the CV of the high-oleic sunflower oil at various frying times. It is clear from the results that the CV of the high-oleic sunflower oil increased significantly with increasing frying time, which may have been caused by the oxidation of triglyceride molecules with unsaturated bonds to produce hydroperoxides under high-temperature conditions. Hydroperoxides are unstable and easily decompose at high temperatures to produce ketones, which increase CV [32].

### 3.4. Determination of Total Oxidation (TOTOX) Value

**Figure 4:**
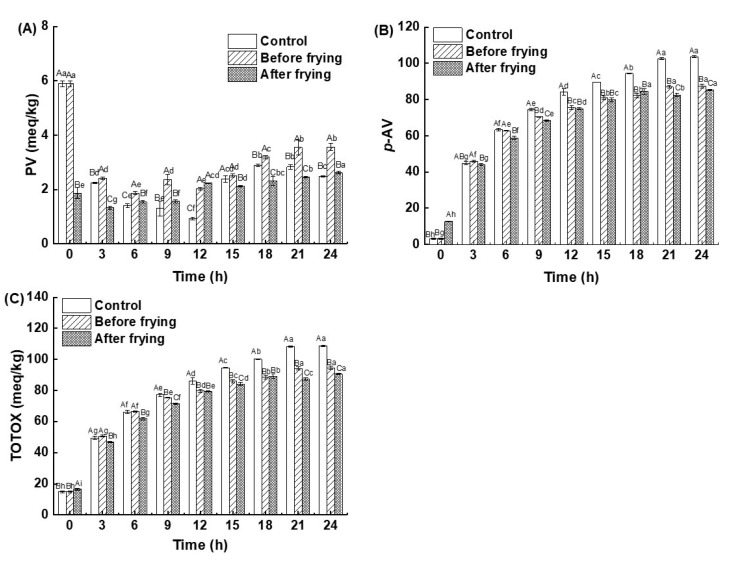
Changes in peroxide values (PVs) (**A**), *p*-anisidine values (*p*-AVs) (**B**), and total oxidation (TOTOX) values (**C**) of high-oleic sunflower oil at different frying times. Note: Different lowercase letters indicated that there were significant differences among the samples prepared with the same deep-frying treatment and different frying times (*p* < 0.05), and different capital letters indicated that there were significant differences among the samples prepared with the same deep-frying time and different deep-frying treatments (*p* < 0.05).

Peroxide value (PV) can reflect the degree of an oil’s oxidation and rancidity. As shown in Figure 4A, the changes in PV of high-oleic sunflower oil at various frying times showed that all PVs were lower than the PV of unheated fresh oil. The PV of high-oleic sunflower oil in the control and deep-fried shrimp groups (before and after frying) first declined and then increased. This might be due to the PV of frying oil having attained a maximum between 0 and 3 h. After 18 h of frying, the PV stabilized owing to a dynamic equilibrium between hydroperoxide formation and decomposition [32]. The *p*-anisidine value (*p*-AV) reflects the amount of aldehydes produced during oil oxidation. The lower the value, the better the oil quality. Figure 4B shows the variation in the *p*-AV of high-oleic sunflower oil at different frying times. It can be seen that the *p*-AVs of the control and treatment groups increased throughout the whole frying process.

TOTOX is a general term for the primary (hydroperoxides) and secondary (aldehydes, ketones, acids, etc.) oxidation products of oil. This may represent the total oxidation of an oil and provide a more thorough assessment of the degradation degree of the oil [33,34,35]. The variation in TOTOX of the high-oleic sunflower oil at various frying times is shown in Figure 4C. It was clear that as the frying time increased, the trends of TOTOX in the control and deep-frying groups were generally consistent with those of the *p*-AVs, with all showing a significant increase followed by a slow increase. After frying for 24 h, the TOTOX of high-oleic sunflower oil achieved its highest values of 108.65 mEq/kg, 94.58 mEq/kg, and 90.51 mEq/kg in the control, before frying, and after frying groups, respectively. Unsaturated fatty acids in oil may spontaneously oxidize at high temperatures to produce hydroperoxides, which are unstable. The decomposition of hydroperoxides results in the formation of aldehydes, ketones, acids, and other compounds [36], thus raising the TOTOX. As the frying time increased, the TOTOX of the deep-fried shrimp group decreased greatly compared to that of the control. This may have been due to the presence of astaxanthin (including free astaxanthin, astaxanthin monoester, and astaxanthin diester) in *L. vannamei* shrimp, which can efficiently scavenge free radicals [25]. It has also been shown that astaxanthin has a stronger antioxidant capacity than both β-carotene and vitamin E [37]. In addition, it has been reported that both boiling and steaming contribute to an increase in astaxanthin levels [38], and it can be inferred that frying also leads to an increase in astaxanthin. The change could be explained by the astaxanthin in the shrimp partially migrating to the frying oil during frying, retarding the oxidation of the oil and thereby reducing TOTOX.

### 3.5. ^1^H NMR Study during Deep-Frying

Changes in the high-oleic sunflower oil during frying of *L. vannamei* shrimp were evaluated using ^1^H NMR spectroscopy. Table 2 shows the fatty acid compositions of frying oil, which were calculated based on the ratio of ^1^H NMR acyl spectra. It was found that the high-oleic sunflower oil contained a high proportion of oleic acid. The oleic acid proportion of fresh high-oleic sunflower oil accounted for 77.42% of the total fatty acids, while the oil used to fry *L. vannamei* shrimp reached 80.05% after 24 h, which is in line with the FAO regulation that the oleic acid proportion of high-oleic sunflower oil should be 75–91% [39]. Furthermore, the amounts of saturated fatty acids and oleic acid increased with longer frying times in both the control and treatment groups, but the amounts of linoleic acid and linolenic acid decreased. This may have been due to breaks in the linkages of linoleic and linolenic acids, which would have changed them into oleic and saturated fatty acids [40].

**Figure 5:**
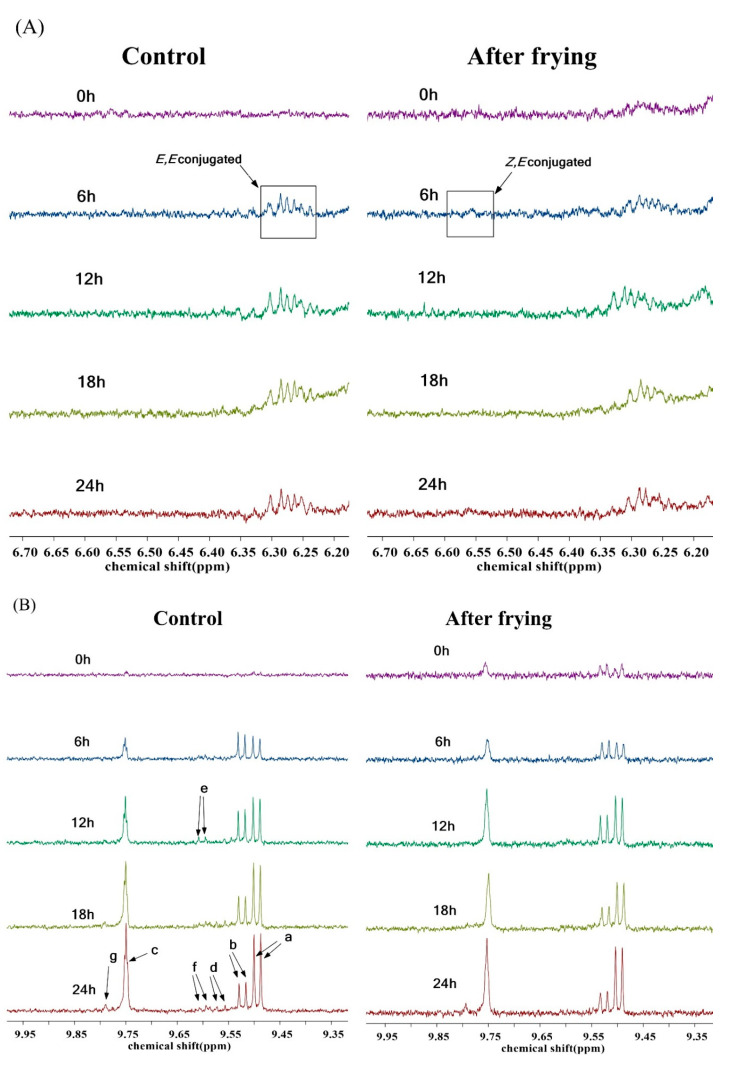

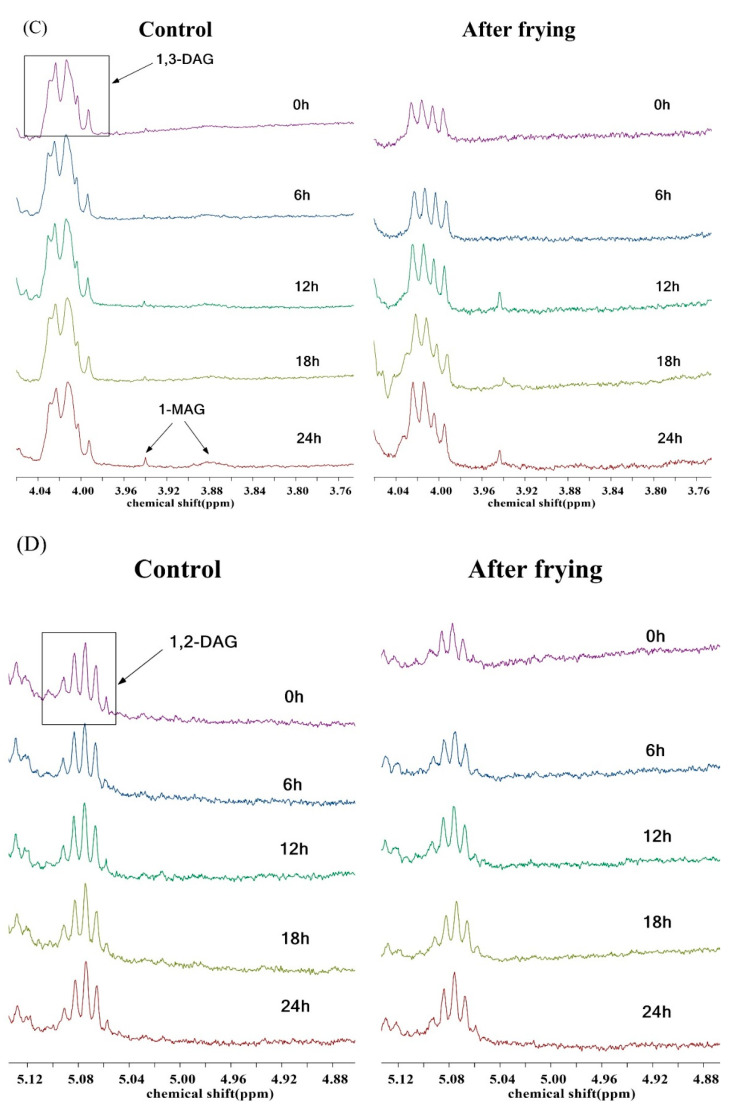
Enlargement of the ^1^H NMR spectral regions: ^1^H NMR spectral signals of *Z,E*- and *E,E*- conjugated hydroperoxides (**A**), ^1^H NMR spectral signals of secondary oxidation products (**B**), ^1^H NMR spectral signals of hydrolysis products (**C**,**D**).

The first oxidation process occurred during the frying process, producing lipid hydroperoxides such as *Z,E*-conjugated and *E,E*-conjugated hydroperoxides. *E,E*-conjugated hydroperoxides have chemical shifts between 6.2 and 6.3 ppm, whereas *Z,E*-conjugated hydroperoxides have chemical shifts between 6.5 and 6.6 ppm [41]. The ^1^H NMR spectra of the control and frying groups during the primary oxidation of high-oleic sunflower oil are shown in Figure 5A. Their contents were calculated based on the NMR spectra and the results are shown in Table 3. The concentration of *E,E*-conjugated hydroperoxides increased slowly with frying, and no *Z,E*-conjugated hydroperoxides were generated in the control. *Z,E*-conjugated hydroperoxides were generated only after 12 h in the frying group, with no significant difference in the intensity in the subsequent frying process. This may be because the *trans* molecular structure has a higher bond energy and higher threshold of formation than that of *cis* molecules [42], so the *Z,E*-conjugated hydroperoxide content was lower than the content of *E,E*-conjugated hydroperoxides. After frying *L. vannamei* shrimp in high-oleic sunflower oil, the major oxidation products exhibited an increasing tendency. During the frying process, lipid hydroperoxides subjected to high temperatures easily enter the secondary oxidation process, further oxidizing to generate aldehydes, ketones, acids, esters, and short-chain carbonyl compounds [43], which are in the range of 9.5–9.8 ppm in the NMR spectrum. It was observed that a bimodal signal was produced by (*E*)-2-alkenals at around 9.50 ppm in the generated compounds (Figure 5(Ba)) and a bimodal signal was generated by (*E,E*)-2,4-alkadienals at around 9.52 ppm (Figure 5(Bb)). The (*E*)-2-alkenals and (*E,E*)-2,4-alkadienals partially overlapped [44]. In addition, the secondary oxidation process produced 4-hydroxy-(*E*)-2-alkenals (Figure 5(Bd)) and 4-hydroperoxy-(*E*)-2-alkenals (Figure 5(Be)), which partially overlapped and therefore co-integrated both compounds in the NMR integration. Furthermore, there were 4,5-epoxy-(*E*)-2-enal (Figure 5(Bf)), 4-oxoalkanes (Figure 5(Bg)), and *n*-alkanes (Figure 5(Bc)). The contents of the secondary oxidation products were calculated from their NMR spectra, as shown in Table 3. It can be seen that the secondary oxidation products showed an increasing trend during frying, while the deep-fried shrimp group showed a faster increase and accumulated more secondary oxidation products than the control, possibly because *L. vannamei* shrimp contain a large percentage of *n*-3 LC-PUFA which may be released into the oil, making it more susceptible to oxidation and increasing oxidation products [45].

**Figure 6:**
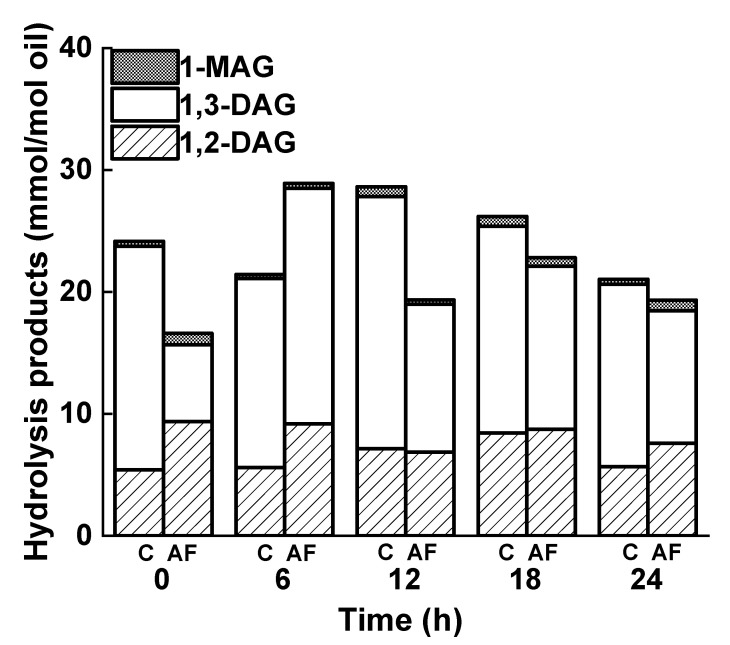
Hydrolysis products of high-oleic sunflower oil during the frying process. C: control, AF: after frying.

During frying, hydrolysis products such as 1,2-DAG, 1,3-DAG, and 1-MAG were also generated (Figure 5C,D) [46,47]. As shown in Figure 6, the quantities of hydrolysis products in the control and deep-fried groups was concentrated in the range of 15–30 mmol/mol oil and reached their maxima at 6–12 h, indicating that the breakage of ester bonds was the most intense at this time [48]. It was also discovered that the hydrolysis products were only minor products of the frying process [49,50]. Furthermore, the majority of the polar components in the frying oil were formed through the oil’s oxidation and polymerization, and these polar compounds were more hazardous than those produced via hydrolysis.

## 4. Conclusions

The oxidation and hydrolysis processes of high-oleic sunflower oil during frying of *L. vannamei* shrimp at 180 °C were examined in this study. The results showed that the chroma ΔE* and a* values of the oil increased and the L* and b* values decreased after high-temperature heating. The AV, *p*-AV, CV, and TOTOX of the oil reached their maxima after continuous frying for 24 h, indicating the accumulation of free fatty acids and oxidation products during the frying process. ^1^H NMR provided information on the fatty acid composition, oxidation, and hydrolysis products of high-oleic sunflower oil. After being used to fry *L. vannamei* shrimp for 24 h, the oleic acid proportion of the oil reached 80.5%; however, the amounts of linoleic and linolenic acids declined with the extension of frying time. Furthermore, the frying process generated large amounts of oxidation products, primarily *Z,E*-conjugated hydroperoxides, (*E*)-2-alkenals, (*E,E*)-2,4-alkadienals, *n*-alkanals, and so on, which accumulated and present potential health risks. These findings provide a theoretical foundation for further research on the utilization of high-oleic oils in practical industrial applications.

## Figures and Tables

**Table 1:** Solid-to-liquid ratio in the fryer.

Batch	Oil Volume (L)	Weight of Shrimp (kg)
1	6.0	0.40
2	5.7	0.38
3	5.4	0.36
4	5.1	0.34
5	4.8	0.32
6	4.5	0.30
7	4.2	0.28
8	3.9	0.26
9	3.6	0.24

**Table 2:** Fatty acid composition of high-oleic sunflower oil during the frying process according to ^1^H NMR (%).

	Time/h	C18:1	C18:2	C18:3	Saturated Fatty Acid
Control	0	77.42 ± 0.23 ^b^	11.8 ± 0.01 ^a^	1.04 ± 0.05 ^a^	9.75 ± 0.18 ^a^
	6	77.43 ± 0.02 ^b^	11.4 ± 0.02 ^a^	0.82 ± 0.01 ^b^	10.34 ± 0.02 ^a^
	12	77.80 ± 1.79 ^ab^	10.23 ± 0.20 ^b^	1.04 ± 0.08 ^a^	10.94 ± 2.07 ^a^
	18	78.97 ± 0.43 ^a^	9.45 ± 0.12 ^c^	1.09 ± 0.15 ^a^	10.49 ± 0.45 ^a^
	24	78.67 ± 0.36 ^ab^	8.95 ± 0.56 ^c^	0.98 ± 0.16 ^a^	11.39 ± 0.04 ^a^
Frying	0	75.78 ± 0.08 ^d^	12.22 ± 0.41 ^a^	0.95 ± 0.02 ^ab^	11.06 ± 0.50 ^a^
	6	76.97 ± 0.36 ^c^	11.17 ± 0.07 ^a^	0.88 ± 0.03 ^c^	10.98 ± 0.31 ^a^
	12	78.57 ± 0.49 ^b^	12.26 ± 2.25 ^a^	0.97 ± 0.05 ^a^	8.20 ± 1.70 ^b^
	18	78.84 ± 0.16 ^b^	10.5 ± 0.94 ^ab^	0.9 ± 0.00 ^bc^	9.76 ± 1.10 ^ab^
	24	80.05 ± 0.19 ^a^	8.79 ± 0.25 ^b^	0.87 ± 0.03 ^c^	10.29 ± 0.03 ^a^

Note: Different lowercase letters indicated that there were significant differences among the samples prepared with the same deep-frying treatment and different frying times (*p* < 0.05).

**Table 3:** Oxidation products of high-oleic sunflower oil during the frying process according to ^1^H NMR (mmol/mol oil).

		Primary Oxidation Products		Secondary Oxidation Products					
	Time/h	*E,E*-Conjugated Hydroperoxides	*Z,E*-Conjugated Hydroperoxides	(*E*)-2-Alkenals	(*E,E*)-2,4-Alkadienals	*n*-Alkanals	4-Hydroxy-(*E*)-2-alkenals + 4-Hydroperoxy-(*E*)-2-alkenals	4,5-Epoxy-(*E*)-2-alkenals	4-Oxoalkanals
Control	0	ND	ND	ND	ND	ND	ND	ND	ND
	6	1.90 ± 0.04 ^b^	ND	1.49 ± 0.02 ^d^	1.52 ± 0.12 ^b^	1.28 ± 0.02 ^d^	0.54 ± 0.02 ^a^	0.25 ± 0.01 ^c^	ND
	12	2.62 ± 0.36 ^ab^	ND	3.28 ± 0.08 ^c^	1.98 ± 0.06 ^a^	3.06 ± 0.10 ^c^	0.56 ± 0.01 ^a^	0.25 ± 0.01 ^c^	0.12 ± 0.02 ^c^
	18	2.62 ± 0.24 ^ab^	ND	5.14 ± 0.06 ^b^	1.96 ± 0.20 ^a^	5.64 ± 0.14 ^b^	0.76 ± 0.03 ^a^	0.60 ± 0.10 ^b^	0.41 ± 0.11 ^b^
	24	3.33 ± 0.52 ^a^	ND	6.05 ± 0.09 ^a^	1.82 ± 0.12 ^a^	6.77 ± 0.17 ^a^	1.15 ± 0.69 ^a^	0.77 ± 0.05 ^a^	0.74 ± 0.26 ^a^
Frying	0	0.74 ± 0.12 ^e^	ND	0.75 ± 0.33 ^d^	0.72 ± 0.19 ^b^	0.47 ± 0.01 ^e^	0.50 ± 0.06 ^c^	0.04 ± 0.03 ^c^	ND
	6	2.08 ± 0.08 ^cd^	ND	2.77 ± 0.04 ^c^	1.46 ± 0.45 ^a^	1.04 ± 0.05 ^d^	1.25 ± 0.12 ^b^	0.23 ± 0.04 ^a^	0.13 ± 0.04 ^b^
	12	3.59 ± 0.07 ^a^	0.55 ± 0.11 ^a^	2.93 ± 0.32 ^c^	1.62 ± 0.02 ^a^	3.44 ± 0.37 ^c^	1.32 ± 0.06 ^b^	0.16 ± 0.03 ^ab^	0.14 ± 0.02 ^b^
	18	2.46 ± 0.88 ^bc^	0.40 ± 0.10 ^a^	3.51 ± 0.18 ^b^	1.38 ± 0.33 ^a^	4.39 ± 0.03 ^b^	1.47 ± 0.05 ^a^	0.12 ± 0.01 ^bc^	0.19 ± 0.12 ^b^
	24	3.41 ± 0.12 ^ab^	0.49 ± 0.01 ^a^	4.97 ± 0.28 ^a^	1.66 ± 0.03 ^a^	5.44 ± 0.31 ^a^	1.24 ± 0.01 ^b^	0.23 ± 0.11 ^a^	0.44 ± 0.13 ^a^

Note: “ND” meant that the substance was not detected. Different lowercase letters indicated that there were significant differences among the samples prepared with the same deep-frying treatment and different frying times (*p* < 0.05).

## Data Availability

The data sets generated during the current study are available from the corresponding author on reasonable request.
